# Single quantum dot-based nanosensor for sensitive detection of 5-methylcytosine at both CpG and non-CpG sites†
†Electronic supplementary information (ESI) available. See DOI: 10.1039/c7sc04813k


**DOI:** 10.1039/c7sc04813k

**Published:** 2017-12-13

**Authors:** Zi-yue Wang, Li-juan Wang, Qianyi Zhang, Bo Tang, Chun-yang Zhang

**Affiliations:** a College of Chemistry, Chemical Engineering and Materials Science , Collaborative Innovation Center of Functionalized Probes for Chemical Imaging in Universities of Shandong , Key Laboratory of Molecular and Nano Probes , Ministry of Education , Shandong Provincial Key Laboratory of Clean Production of Fine Chemicals , Shandong Normal University , Jinan 250014 , China . Email: cyzhang@sdnu.edu.cn ; Email: tangb@sdnu.edu.cn ; Fax: +86-0531-82615258 ; Tel: +86-0531-86186033; b Nantou High School Shenzhen , Shenzhen , 518052 , China

## Abstract

DNA methylation is an important epigenetic modification in human genomes. Herein, we develop a single quantum dot (QD)-based nanosensor for sensitive detection of DNA methylation at both CpG and non-CpG sites using tricyclic ligation chain reaction (LCR)-mediated QD-based fluorescence resonance energy transfer (FRET). We design two sets of DNA probes (X and Y, X′ and Y′) for methylated DNA assay. In the presence of thermostable DNA ligase, probes X and Y may adjacently hybridize with the methylated DNA to obtain the ligated XY products which may function as the templates for probes X′ and Y′ to generate the X′Y′ products. The resultant X′Y′ products may in turn act as the templates to ligate probes X and Y for the generation of XY products, consequently inducing tricyclic LCR amplification under thermal denaturation conditions to generate a large number of XY products. The subsequent hybridization of XY products with the capture and reporter probes results in the formation of sandwich hybrids which may assemble on the 605QD surface to obtain 605QD–oligonucleotide–Cy5 nanostructures, inducing efficient FRET from the 605QD to Cy5 and the emission of Cy5. This nanosensor can detect DNA methylation at single 5-methylcytosine (5-mC) resolution with a detection limit of as low as 1.0 aM and a large dynamic range of 7 orders of magnitude. Moreover, this nanosensor can distinguish as low as a 0.01% methylation level, and it can detect DNA methylation in human lung cancer cells as well, holding great potential for accurate epigenetic evaluation and early cancer diagnosis.

## Abstract 2

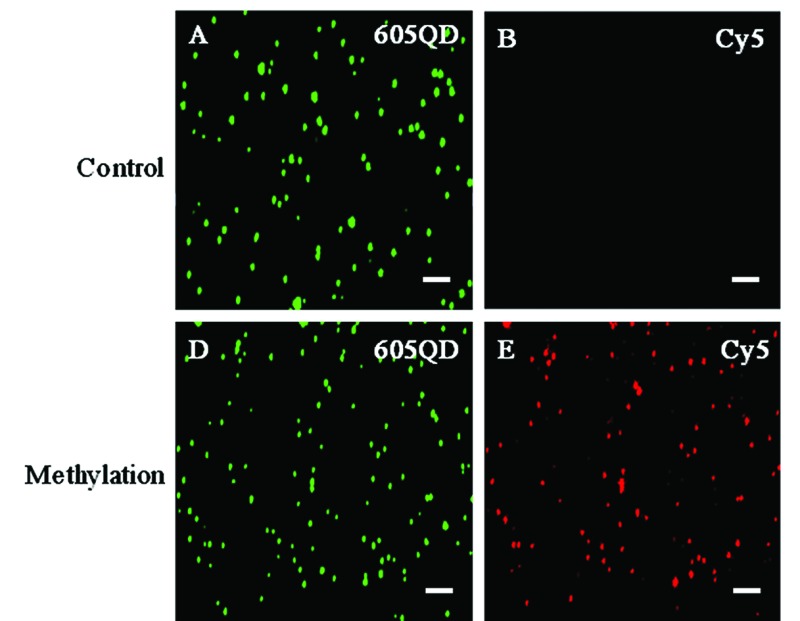
We develop a single quantum dot (QD)-based nanosensor for sensitive detection of DNA methylation at both CpG and non-CpG sites.

## Introduction

DNA methylation, a highly characterized epigenetic modification in human genomes, is frequently occurring at the carbon-5 position of cytosine residues in cytosine/guanine dinucleotides (CpG) islands through the addition of a methyl group to the carbon 5 of cytosine with the catalysis of DNA methyltransferase.^1^,^2^ Each CpG island may own several tens to hundreds of CpG repetitions, constituting the main promoter regions of genes.^3^,^4^ Hypermethylation of CpG islands in promoter regions may lead to the deregulation of a diverse array of functions in human cells, including DNA replication, DNA repair, gene transcription, X chromosome inactivation, genomic imprinting, and cellular differentiation.^5^–^7^ Aberrant DNA methylation patterns are closely related to various genetic diseases and cancers, such as prostate, colon, lung, liver, kidney, breast, cervix, thyroid, retinoblastoma, and hematologic cancers.^8^–^13^ Recent researches demonstrate that DNA methylation (*i.e.* CpA, CpT and CpC) in non-CpG sites plays important roles in gene expression regulation.^14^,^15^ High level of non-CpG methylation within gene bodies in the genome is correlated with the level of expression of the corresponding genes in human mature oocyt.^16^ Moreover, 5-methylcytosine (5-mC) followed by a nucleotide other than guanine (mCH, where H = A or T or C) may regulate neuronal gene expression through its recognition by methyl CpG binding protein 2 (MeCP2) in the neurological disorder Rett syndrome.^17^ Consequently, DNA methylation may function as both a hallmark of large scale epigenetic events and a potential biomarker of various diseases, and the accurate quantification of DNA methylation at both CpG and non-CpG sites is not only of high importance to early diagnosis, prognosis, and therapy of cancers, but also of great significance to biochemical research and drug development.

So far, a variety of methods have been developed for DNA methylation assay. Conventional methods are mainly based on the polymerase chain reaction (PCR), such as chemical DNA sequencing in combination with ligation-mediated PCR (LM-PCR),^18^ methylation sensitive arbitrary primed PCR (MS-AP-PCR),^19^ methylation-specific PCR (MS-PCR),^20^ fluorescence-based real-time PCR (Fb-RT-PCR),^21^ and methylation-specific quantum dot-based fluorescence resonance energy transfer (MS-qFRET).^22^ However, these PCR-based methods usually involve complicated operating procedures (*e.g.* specific reaction temperature and cycle numbers) with some limitations. The LM-PCR requires the pretreatment of nonmethylated cytosine, gel sequencing, and laborious operations.^18^ The MS-AP-PCR employs restriction enzymes for methylation analysis with the requirement of specific restriction sites and radioactive labeling.^19^ The MS-PCR may precisely map the methylation patterns in CpG islands, but it only provides qualitative data instead of quantitative data.^20^ The Fb-RT-PCR may provide quantitative and real-time results, but the involvement of double-labeled TaqMan probes make it expensive and susceptible to contamination.^21^,^23^ The MS-qFRET facilitates straightforward detection of DNA methylation, but the end-labeling of all MSP primers is inconvenient and expensive.^22^ Alternatively, several PCR-free methods have been developed for DNA methylation assay.^24^–^28^ Quartz crystal microbalance (QCM) may measure DNA methylation with low DNA sample consumption, but it requires the enrichment of methylated DNAs by PCR and the immobilization of thiol-functionalized DNA probes on the gold surfaces of QCM chips.^24^ The combination of methylated DNA precipitation with luciferase-fused zinc finger assay (MELZA) enables chemiluminescent measurement of DNA methylation in the androgen receptor (AR) promoter region,^25^ but the constructions of glutathione S-transferases (GST)-tagged methyl CpG-binding domain (MBD) protein and GST-tagged zif268-luciferase are cumbersome with the requirement of professional operation skills. Graphene oxide (GO)-based electrochemical method enables site-specific detection of DNA methylation,^26^ but the synthesis of GO sheets, modification of thionine on the GO surface, deposition of gold nanoparticles (AuNPs) on glassy carbon (GC) electrode surface, modification of DNA probes on AuNP-covered electrode and conjugation of thionine/GO involve complicated experimental procedures. Hyperbranched rolling circle amplification (HRCA)^27^ and nicking enzyme signal amplification (NESA)-based fluorescent methods^28^ enable sensitive detection of DNA methylation, but they suffer from nonspecific amplification induced by the preferential binding of SYBR green I to the GC-rich DNA sequences^29^ and the combinational use of nickase and DNA polymerase.^30^ Notably, due to the requirement of either specific recognition sequences for restriction endonucleases (*e.g.* Hpa II, Msp I, BstUI or TaqI)^19^,^21^,^24^,^26^ and chemical cleavage^18^ or specific sequences with sufficient 5-mC sites for primer design,^20^,^22^,^25^,^27^,^28^ all the above methods are only suitable for the detection of DNA methylation at CpG sites instead of non-CpG sites. So far, there has been only one reported method of ligation-depended PCR capable of detecting DNA methylation at non-CpG sites.^31^ Actually, ligation-depended PCR can simultaneously detect multisite 5-mC including non-CpG sites, but it is time-consuming with poor sensitivity due to the involvement of denatured polyacrylamide electrophoresis gel for the analysis of reaction products.^31^ Therefore, the development of simple and rapid methods for sensitive detection of DNA methylation at both CpG and non-CpG sites is highly desirable.

Ligase chain reaction (LCR) uses thermostable DNA ligase to repeatedly ligate adjacently hybridized DNA probes, and may exponentially amplify target DNA.^32^,^33^ In comparison with other nucleic acid amplification approaches (*e.g.* PCR,^34^ rolling circle amplification,^27^ and exponential isothermal amplification^28^), LCR has distinct advantages: (1) thermal cycles of ligation reaction enables the exponential amplification of target oligonucleotides with an attomolar sensitivity;^35^ (2) high fidelity thermostable DNA ligase exhibits exceptional specificity on a single base discrimination;^33^ (3) no complicated DNA polymerization reaction is required. Therefore, LCR has become a simple and robust alternative platform for nucleic acid amplification. Recently, QD-based FRET in combination with single-molecule detection has attracted more and more attention in biomedical fields,^36^–^41^ and they have been widely applied for sensitive detection of various biomolecules including DNAs, RNAs, enzymes, small molecules and cancer cells.^37^,^39^,^42^ Herein, we develop a new method for sensitive detection of DNA methylation at both CpG and non-CpG sites using tricyclic LCR-mediated QD-based FRET. Due to high specificity of ligation reaction mediated by thermostable DNA ligase, high amplification efficiency of tricyclic LCR, and high sensitivity of single-molecule detection, the proposed method can detect DNA methylation at single 5-mC resolution with a detection limit of 1.0 aM and a large dynamic range of 7 orders of magnitude. This method can distinguish as low as a 0.01% methylation level from a mixture of methylated and unmethylated DNA and can accurately quantify DNA methylation level in even one single cancer cell.

## Results and discussion

**Scheme 1:**
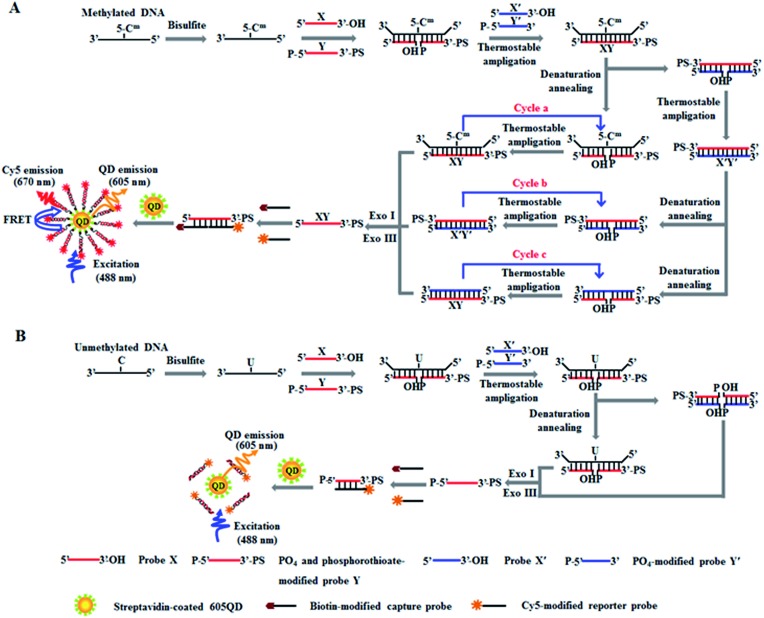
Scheme illustration of DNA methylation assay based on tricyclic LCR-mediated QD-based FRET. (A) The detection of methylated DNA involves three steps: (1) the conversion of cytosines in target DNA to uracils by bisulfite treatment, (2) DNA methylation-actuated tricyclic LCR amplification including cycles a, b and c, and (3) the QD-based FRET measurement by TIRF-based single-molecule imaging. (B) In the absence of methylated DNA, all cytosines in target DNA are converted to uracils, and thus no tricyclic LCR amplification is actuated and no FRET between the 605QD donor and the Cy5 acceptor occurs.

The principle of DNA methylation assay is illustrated in Scheme 1. This assay involves three steps: (1) the complete conversion of cytosines in target DNA to uracils through bisulfite treatment, (2) DNA methylation-actuated tricyclic LCR amplification, and (3) QD-based FRET measurement by TIRF-based single-molecule imaging. We designed a 46-nt target DNA with one single 5-mC that is 22-base away from the 5′ end (Table 1). Unlike multisite 5-mC in CpG islands, methylated DNA in non-CpG region is usually emerging in the form of a single 5-mC site. In theory, the proposed method with the capability of detecting one single 5-mC may be applied for the detection of 5-mC sites at both CpG islands and single 5-mC site at non-CpG region. We designed two sets of DNA probes (X and Y, X′ and Y′) for the recognition of target DNA and the subsequent initiation of LCR amplification. The probes X and Y (Scheme 1, red color) are complementary to the sequence of target DNA, with probe Y being modified with phosphate group (PO_4_) at the 5′ end for ligation reaction and phosphorothioate at the 3′ end for the prevention of Exo I and III digestion. Probes X′ and Y′ (Scheme 1, blue color) are complementary to the sequences of probes Y and X, respectively, with probe Y′ being modified with PO_4_ group at the 5′ end for the ligation with X′ probe to generate the ligation template X′Y′. The 3′-biothylated capture probe is perfectly complementary to the sequence of probe X, while the 5′-Cy5-labeled reporter probe is partly complementary to the sequence of probe Y. Prior to DNA methylation assay, target DNA is subjected to bisulfite treatment to completely convert cytosines to uracils, with the methylated cytosines remaining unchanged.^43^ Notably, this assay involves three cycles (*i.e.* cycles a, b and c). In cycle a, probes X and Y hybridize adjacently with target DNA at the position of 5-mC to obtain XY products after ligation by thermostable Ampligase, and this reaction may be cycled under thermal denaturation condition (Scheme 1A, cycle a). In cycle b, the XY products obtained in cycle a may function as the ligation templates for probes X′ and Y′ to obtain the X′Y′ products after ligation by thermostable Ampligase, and this reaction may be cycled under thermal denaturation condition (Scheme 1A, cycle b). In cycle c, the X′Y′ products obtained in cycle b may in turn function as the new ligation templates for free probes X and Y to obtain the XY products after ligation by thermostable Ampligase, and this reaction may be cycled under thermal denaturation condition (Scheme 1A, cycle c). Importantly, the XY products obtained in cycle c may function as the ligation templates for probes X′ and Y′ to initiate cycles of hybridization–ligation–denaturation under cyclic thermal denaturation condition, inducing tricyclic LCR amplification (Scheme 1A, cycles a, b and c) for exponential amplification and generating large numbers of XY products. Subsequently, Exo I and III are added into the reaction system to digest the excess probes X, X′, Y′ and X′Y′ products, but the XY products cannot be digested because the phosphorothioate modification at the 3′ end of probes Y can efficiently prevent the digestion of Exo I and III.^44^ The remaining XY products may hybridize with the capture and reporter probes to obtain the sandwich hybrids which may assemble on the surface of 605QDs to form the 605QD–oligonucleotide–Cy5 nanostructures through specific biotin–streptavidin interaction (Scheme 1A). Due to the spatial proximity between 605QD and Cy5 in 605QD–oligonucleotide–Cy5 nanostructure, efficient fluorescence resonance energy transfer (FRET) may occur between the 605QD donor and the Cy5 acceptor, leading to the emission of Cy5. Through simply counting the Cy5 molecule by TIRF-based single-molecule imaging, the DNA methylation level can be accurately quantified. In contrast, in the absence of methylated DNA, no ligation reaction occurs because the guanine–uracil mismatch is exactly at the ligation site. As a result, neither XY products nor the 605QD–oligonucleotides–Cy5 nanostructures can be formed, and no Cy5 emission from FRET is observed (Scheme 1B).

**Table 1:** Sequences of the oligonucleotides^*a*^

Note	Sequences (5′–3′)
Methylated DNA	TTG ACT CTG TGG AGT CCT GCA CGA GAC TAG TCA GTA CAC TGC AAG T
Unmethylated DNA	TTG ACT CTG TGG AGT CCT GCA CGA GAC TAG TCA GTA CAC TGC AAG T
Probe X	TAC TGA CTA GTC TCG
Probe Y	P-TGC AGG ACT CCA CAG *AG*
Probe X′	CTC TGT GGA GTC CTG CA
Probe Y′	P-CGA GAC TAG TCA GTA
Capture probe	CGA GAC TAG TCA GTA-biotin
Reporter probe	TGA CTC TGT GGA GTC C*T*G CC
Synthesized reporter probe	TGA CTC TGT GGA GTC C*T*G CCC GAG ACT AGT CAG TA-biotin

^*a*^In methylated DNA, the italicized “*C*” base indicates the C5-methylcytosine (5-mC). In probe Y, the “P” indicates the phosphate group (PO_4_) modification at the 5′ end, and the italicized “*AG*” bases indicate the phosphorothioate (PS) modification at the 3′ end. In probe Y′, the “P” indicates the PO_4_ modification at the 5′ end. In capture probe, the 3′ end is modified with a biotin. In reporter probe, the italicized “*T*” base is modified with a Cy5 molecule. In the synthesized reporter probe, the italicized “*T*” base is modified with a Cy5 molecule, and the 3′ end is modified with a biotin.

**Fig. 1:**
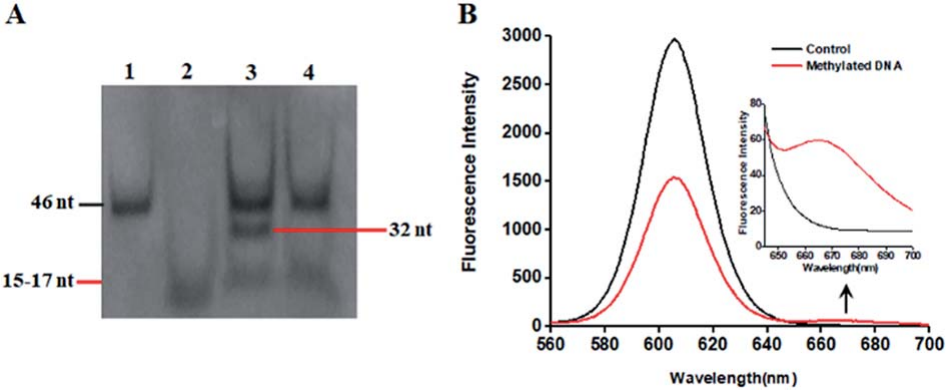
(A) Denaturating PAGE analysis of reaction products. Lane 1 is the synthesized methylated DNA target. Lane 2 is the DNA probes X and Y′ (15 nt), X′ and Y (17 nt). Lane 3 is the reaction products in the presence of methylated DNA. Lane 4 is the reaction products in the absence of methylated DNA. (B) Variance of 605QD and Cy5 fluorescence spectra in the absence (control group, black curve) and presence of methylated DNA (red curve). The inset shows the magnified fluorescence spectra from 660 to 700 nm. The concentration of methylated DNA is 10 nM, and the concentration of each DNA probes X, Y, X′ and Y′ is 1.0 × 10^–6^ M.

This proposed method is dependent on the successful ligation of DNA probes (X and Y, X′ and Y′) in the presence of methylated DNA to trigger the tricyclic LCR amplification. To investigate whether single 5-mC may induce the ligation reaction under the cyclic thermal denaturation condition, we used denaturating gel electrophoresis to analyze the ligation products. As shown in Fig. 1A, in the presence of 10 nM target DNA containing one 5-mC, three bands of 46 nt, 32 nt and 15–17 nt are observed (lane 3), with the 46 nt band indicating the methylated DNA target (lane 1), and the 15–17 nt band indicating the unligated probes X and Y′ (15 nt), X′ and Y (17 nt) (lane 2), and the 32 nt band is exactly the characteristic band of ligated XY and X′Y′ products (lane 3). While in the absence of methylated DNA target, only two bands of 46 nt and 15–17 nt are shown (lane 4), but no characteristic bands of ligated XY and X′Y′ products (32 nt) are detected, implying that the single mismatch of guanine–uracil (G–U) cannot induce the ligation reaction. These results demonstrate that (1) the methylated DNA can successfully induce the ligation of DNA probes and that (2) the single 5-mC-actuated LCR exhibits excellent specificity (no extra bands shown in lane 4).^45^ We further monitored the variance of 605QD and Cy5 fluorescence signals with the addition of capture probes, reporter probes and streptavidin-coated 605QDs into the reaction system (Fig. 1B). In the control group without methylated DNA, only the 605QD fluorescence signal is detected (Fig. 1B, black curve), but no Cy5 fluorescence signal is observed. While in the presence of methylated DNA, the decrease of 605QD fluorescence signal and the increase of Cy5 fluorescence signal are simultaneously detected (red curve), indicating efficient FRET from the 605QD donor to Cy5 acceptor in the 605QD–oligonucleotides–Cy5 nanostructure formed by the hybridization of capture and reporter probes with the XY products which result from single 5-mC-actuated tricyclic LCR. Both gel electrophoresis (Fig. 1A) and fluorescence measurements (Fig. 1B) demonstrate that the proposed method is feasible for the detection of single 5-mC site.

**Fig. 2:**
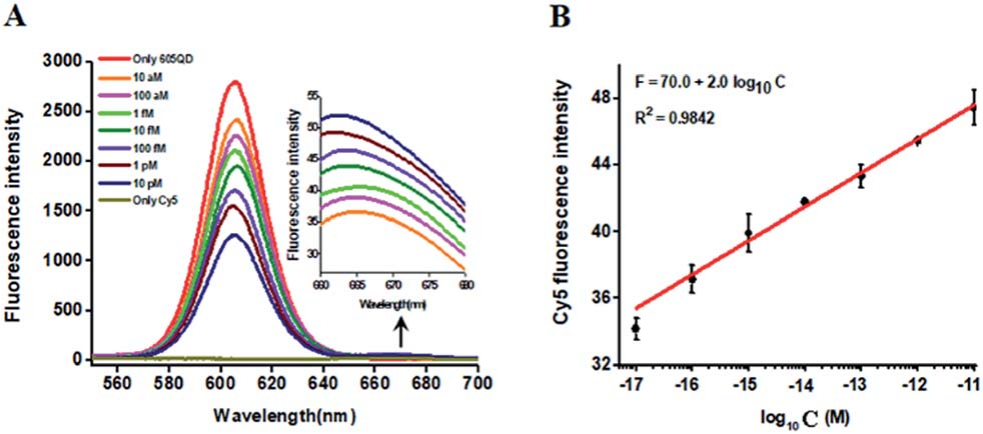
(A) Variance of fluorescence spectra with different-concentration methylated DNA. The inset shows the magnified fluorescence spectra from 660 to 700 nm. (B) Linear relationship between the Cy5 fluorescence intensity and the logarithm of methylated DNA concentration. The concentration of each DNA probes X, Y, X′ and Y′ is 1.0 × 10^–6^ M. The concentration of capture probe is 2.0 × 10^–7^ M, the concentration of reporter probe is 2.0 × 10^–7^ M, and the concentration of 605QD is 8.3 nM. Error bars show the standard deviations of three experiments.

In this research, we selected the 605 nm emitting QD (605QD) and cyan dye Cy5 as the energy donor and the energy acceptor, respectively, based on following three reasons: (1) high quantum yield of 605QD (∼0.6) and high extinction coefficient of Cy5 (∼250 000 M^–1^ cm^–1^), (2) no direct excitation of Cy5 at the excitation wavelength of 488 nm and no cross talk between the emission spectrum of 605QD and that of Cy5, and (3) the capability of assembling multiple oligonucleotides on the surface of a single 605QD for improved FRET efficiency.^42^ In theory, the average distance from the 605QD to Cy5 in the 605QD–oligonucleotides–Cy5 nanostructure is 12.3 nm (the distance between two adjacent bases is 0.34 nm for dsDNA and the radius of a streptavidin-coated 605QD is 0.5–7.5 nm),^46^ within the efficient distance of FRET (2*R*_0_ = 13.9 nm).^42^ We further monitored the variance of FRET efficiency in response to different-concentration methylated DNA under the optimally experimental conditions (see ESI, Fig. S1–S4†). As shown in Fig. 2A, the 605QD fluorescence intensity decreases monotonically with the increasing concentration of methylated DNA, accompanied by the increase of Cy5 fluorescence intensity correspondingly, suggesting the improved FRET efficiency induced by single 5-mC-actuated tricyclic LCR amplification-mediated formation of the 605QD–oligonucleotides–Cy5 nanostructures. Moreover, a good linear correlation is obtained between the Cy5 fluorescence intensity and the logarithm of methylated DNA concentration in the range from 1 × 10^–17^ to 1 × 10^–11^ M (Fig. 2B). The corresponding equation is *F* = 70.0 + 2.0 log_10_ *C* (*R*^2^ = 0.9842), where *F* represents the Cy5 fluorescence intensity and *C* represents the methylated DNA concentration (M). The detection limit is determined to be 1.0 × 10^–17^ M. This result demonstrates that the Cy5 fluorescence signal of the proposed method can be used for DNA methylation assay.

**Fig. 3:**
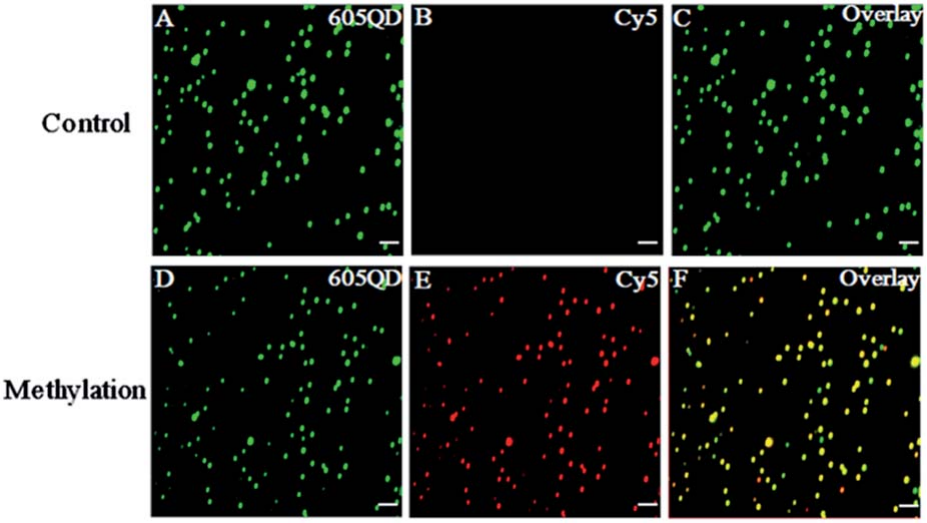
Fluorescence images of 605QD and Cy5 obtained by TIRF-based single-molecule detection in the absence (A–C) and presence (D–F) of methylated DNA. The fluorescence signal of 605QD is shown in green (A and D), and the fluorescence signal of Cy5 is shown in red (B and E), and the colocalization of the 605QD and Cy5 fluorescence signals is shown in yellow (C and F). The concentration of methylated DNA is 1.0 × 10^–11^ M. The concentration of each DNA probes X, Y, X′ and Y′ is 1.0 × 10^–6^ M. The concentration of 605QD is 8.3 nM. The scale bar is 2 μm.

We used total internal reflection fluorescence (TIRF) microscopy to detect methylated DNA at the single-molecule level.^47^ In this assay, the largest separation distance between the 605QD and Cy5 in the 605QD–oligonucleotide–Cy5 nanostructure is calculated to be 27.3 nm (the radius of a streptavidin functionalized 605QD is 5.0–7.5 nm,^42^ and the separation distance between 605QD and Cy5 is 12.3 nm), within the excitation field of TIRF (<100 nm).^42^ As shown in Fig. 3, in control group, only the fluorescent signals of the 605QD donor are detected (Fig. 3A, green color), but no fluorescent signal of the Cy5 acceptor is observed (Fig. 3B, red color), suggesting no direct excitation of Cy5 acceptors at the excitation wavelength of 488 nm and no leakage of 605QD spectrum into the Cy5 spectrum. In contrast, the fluorescent signals of both the 605QD (Fig. 3D) and Cy5 (Fig. 3E) are simultaneously detected in the presence of methylated DNA, with distinct yellow color (Fig. 2F) indicating the perfect colocalization of the 605QD and Cy5, demonstrating efficient FRET from the 605QD to Cy5 in the presence of methylated DNA. In addition, near-zero background Cy5 fluorescence signal is observed in the control group without methylated DNA (Fig. 3B), suggesting high specificity of the proposed method.

**Fig. 4:**
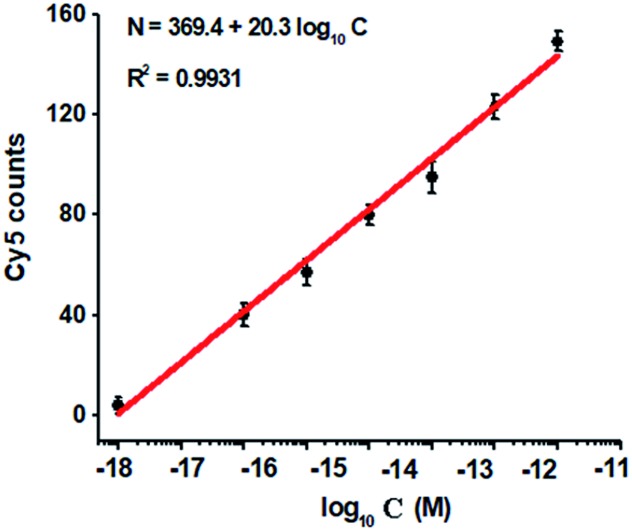
Variance of Cy5 counts with different-concentration methylated DNA. The Cy5 counts exhibit a linear correlation with the logarithm of methylated DNA concentration in the range from 1 × 10^–18^ to 1 × 10^–11^ M. The concentration of each DNA probes X, Y, X′ and Y′ is 1.0 × 10^–6^ M. The concentration of 605QD is 8.3 nM. Error bars show the standard deviation of three experiments.

To investigate the detection sensitivity of the proposed method, we measured the variance of Cy5 counts in response to different-concentration methylated DNA. As shown in Fig. 4, the Cy5 counts increase in a concentration-dependent manner. In logarithmic scale, the Cy5 counts exhibit a good linear correlation with the concentration of methylated DNA over a large range of 7 orders of magnitude from 1.0 × 10^–18^ to 1.0 × 10^–11^ M (Fig. 4), with 1 order of magnitude wider than that of ensemble measurement (Fig. 2). The regression equation is *N* = 369.4 + 20.3 log_10_ *C* with a correlation coefficient of 0.9931, where *N* represents the Cy5 counts and *C* represents the concentration of methylated DNA (M), respectively. The detection limit is determined to be 1.0 × 10^–18^ M (Fig. 4), with 10-fold improvement compared with that of ensemble measurement (Fig. 2), suggesting the improved sensitivity of TIRF-based single-molecule detection. Notably, the sensitivity of the proposed method has improved by as much as 5 orders of magnitude compared with that of nicking enzyme signal amplification (NESA)-based fluorescent assay,^28^ and 2 orders of magnitude compared with that of hyperbranched rolling circle amplification (HRCA),^27^ and 20-fold as compared with that of ligation-depended PCR.^31^ The improved sensitivity may be attributed to following three factors: (1) the high specificity of ligation reaction mediated by the high fidelity thermostable Ampligase, (2) the high amplification efficiency of tricyclic LCR, and (3) the high sensitivity of TIRF-based single-molecule detection.

**Fig. 5:**
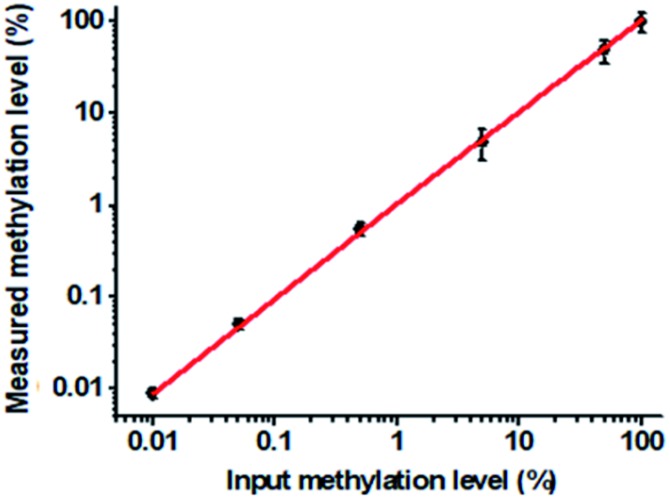
Correlation between the measured and the actual input methylation level in the mixtures of methylated and unmethylated DNA. The concentration of each DNA probes X, Y, X′ and Y′ is 1.0 × 10^–6^ M. The concentration of 605QD is 8.3 nM. Error bars show the standard deviation of three experiments.

To verify the feasibility of the proposed method for accurate measurement of DNA methylation level in the mixture, we prepared a series of artificial mixtures by mixing methylated and unmethylated DNA at different ratios. The measured methylation level is calculated on the basis of eqn (1).1
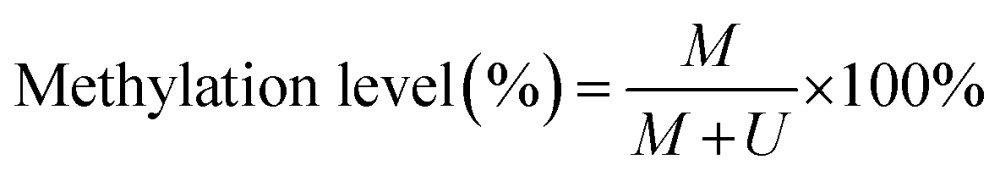
where *M* is the quantity of methylated DNA measured by the proposed method and *U* is the quantity of unmethylated DNA. When the amount of methylated DNA increases in the mixture, the 605QD fluorescence signal decreases correspondingly as a result of FRET from the 605QD donor to the Cy5 acceptor (see ESI, Fig. S5†), and the Cy5 counts enhance with the increasing methylated DNA ratio in the mixture (Fig. 5). Notably, a linear relationship is obtained between the measured methylation level and the input methylation level. The regression equation is *Y* = 1.047*X* – 0.001 with a correlation coefficient of 0.9966, where *Y* is the measured methylation level (%) and *X* is the input methylation level (%), respectively. Importantly, this method can even distinguish as low as a 0.01% methylation level, superior to most of the reported methods for DNA methylation assay, including MS-qFRET (1%),^22^ MS-PCR (0.1%),^20^ and NESA-based methods (0.1%).^28^ The high discrimination capability may be ascribed to following four factors: (1) a single-base discrimination of the high fidelity thermostable Ampligase, (2) the high specificity of tricyclic LCR, (3) the high efficiency of Exo I and III-catalyzed digestion, and (4) the high signal-to-noise ratio of single-molecule detection.

**Fig. 6:**
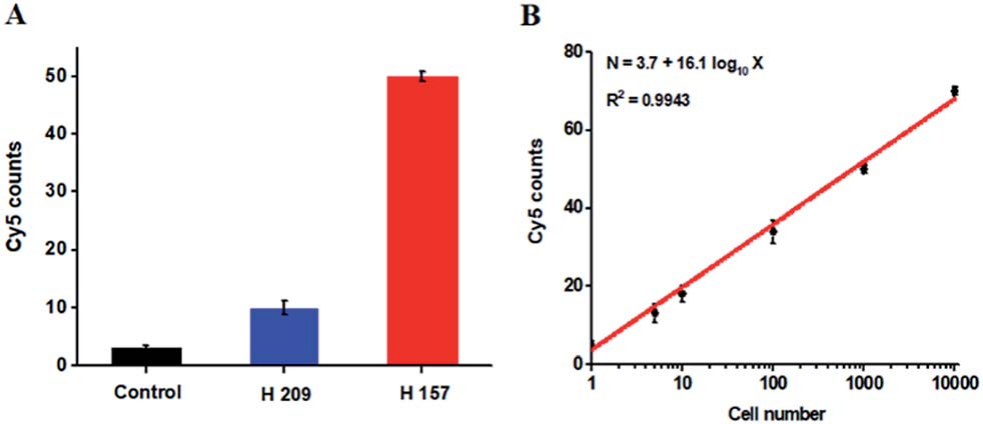
(A) Measurement of Cy5 counts in the presence of lysis buffer (control group, black column), 1000 H209 cells (blue column), and 1000 H157 cells (red column), respectively. (B) Linear relationship between the Cy5 counts and the logarithm of H157 cell number in the range from 1 to 10 000 cells. The concentration of each DNA probes X, Y, X′ and Y′ is 1.0 × 10^–6^ M. The concentration of 605QD is 8.3 nM. Error bars show the standard deviations of three experiments.

The hypermethylation of CpG island in pl6 tumor suppressor gene has strong association with the complete loss of gene transcription in various cancers.^48^ To further investigate the feasibility of the proposed method for quantitative detection of DNA methylation in CpG sites, we designed two sets of DNA probes (see ESI, Table S1†) to detect DNA methylation in CpG island in pl6 tumor suppressor genes from human lung cancer cell lines. We used H157 cells (nonsmall-cell lung cancer cell line) and H209 cells (small-cell lung cancer cell line) as the models of methylated DNA and unmethylated DNA, respectively. Genomic DNA was extracted from the above cancer cells, followed by Pst I and BstE II digestion to avoid DNA supercoiling and decircularization during the heating process.^27^ The resultant DNA fragments were pretreated with bisulfite prior to DNA methylation assay. As shown in Fig. 6A, a high Cy5 signal is obtained in H157 cells (Fig. 6A, red column), but no significant Cy5 signal is observed in H209 cells (Fig. 6A, blue column) and control group with only lysis buffer (Fig. 6A, black column), respectively, consistent with the previous report that the CpG sites are highly methylated in H157 cells but unmethylated in H209 cells.^20^ Moreover, the Cy5 counts improve with the increasing number of H157 cells (Fig. 6B), with a linear correlation being obtained between the Cy5 count and the logarithm of H157 cell number in the range from 1 to 10 000 cells. The corresponding equation is *N* = 3.7 + 16.1 log_10_ *X* (*R*^2^ = 0.9943), where *N* represents the Cy5 counts and *X* represents the number of H157 cells, respectively. The detection limit is determined to be 1 cancer cell, suggesting the feasibility of the proposed method for sensitive detection of DNA methylation level in CpG islands from cancer cells. These results (Fig. 6) are consistent with the measurement of fluorescence spectra of same cellular samples (see ESI, Fig. S6†). Above all, our results clearly demonstrate that the combination of LCR with single-molecule imaging enables accurate detection of not only single 5-mC at non-CpG sites (Fig. 4) but also multiple 5-mC at CpG sites with high sensitivity (Fig. 6B).

## Conclusions

In conclusion, we have developed a simple and rapid method for sensitive detection of DNA methylation at both CpG and non-CpG sites on the basis of single 5-mC-actuated tricyclic LCR-mediated QD-based FRET. Taking advantage of the high specificity of ligation reaction mediated by high fidelity thermostable Ampligase, the high amplification efficiency of tricyclic LCR and the high sensitivity of single-molecule detection, this method can detect DNA methylation at single 5-mC resolution with a detection limit of as low as 1.0 aM and exhibits a large dynamic range of 7 orders of magnitude. This detection sensitivity of this method has improved by 5 orders of magnitude compared with those of NESA-based fluorescent assay,^28^ 2 orders of magnitude compared with HRCA-based fluorescent assay,^27^ and 20-fold compared with the ligation-depended PCR assay.^31^ Unlike the established approaches for DNA methylation assay (see ESI, Table S2†),^18^–^22^,^24^,^26^–^28^ this method involves no specific regions with multisite 5-mC for designing primers, no restriction enzymes for recognizing specific 5-mC sites, no polymerase/endonuclease for amplification reaction (*e.g.*, PCR, HRCA, and NESA), no radioactive materials for labeling the reaction products, no complicated biochemical reactions for synthesizing particular materials, and no enrichment of methylated DNA. Especially, this is a homogeneous assay with the involvement of only monotonous ligation reaction mediated by one thermal DNA ligase, without the requirement of multiple enzymes and complicated reactions, endowing this method with simplicity, rapidity and convenience. Importantly, this method can distinguish as low as a 0.01% methylation level from the mixtures and it can be applied for accurate detection of DNA methylation levels even in one single cancer cell, holding great potential in epigenetic modification research and the early diagnosis of methylation-related human diseases.

## Experimental section

### Chemicals and materials

All oligonucleotides were synthesized and HPLC purified by Sangon Biotechnology Co. Ltd. (Shanghai, China). Thermostable Ampligase was obtained from Epicenter Technologies (Madison, WI, U.S.A.), exonuclease I (Exo I) and exonuclease III (Exo III) were purchased from New England Biolabs (Ipswich, MA, U.S.A.), magnesium chloride (MgCl_2_), ammonium sulfate (NH_4_)_2_SO_4_, bovine serum albumin (BSA), trolox, glucose oxidase, d-glucose, and catalase were obtained from Sigma-Aldrich Company (St. Louis, MO, U.S.A.). The streptavidin-coated quantum dots with the maximum emission at 605 nm (Qdot 605 ITK) were obtained from Life Technologies (Eugene, Oregon, U.S.A.). All other reagents were of analytical grade and used just as received without further purification. The ultrapure water was prepared by a Millipore filtration system (Millipore, Milford, MA, U.S.A.).

### Bisulfite treatment of DNA

Bisulfite treatment of DNA was performed according to the reported method.^43^ First, 2 μg of DNA was denatured in 0.35 M NaOH at 42 °C for 30 min. Bisulfite reaction was carried out in 3.2 M sodium bisulfite and 0.5 mM hydroquinone (both were freshly prepared) at 50 °C for 16–18 h. Then DNA was recovered with a desalting column (DNA cleanup system, Promega Inc., U.S.A.) and the modification was completed in 0.3 M NaOH at 37 °C for 15 min, followed by neutralization with ammonium acetate, precipitation with ethanol, and drying. The resulting DNA was resuspended in water and used immediately or stored at –20 °C.

### Tricyclic LCR amplification

The LCR reaction was performed in 20 μL of mixture solution containing 20 mM Tris–HCl (pH 8.3), 25 mM KCl, 10 mM MgCl_2_, 0.5 mM nicotinamide adenine dinucleotide (NAD), 0.01% triton X-100, 1 μM probe X, 1 μM probe Y, 1 μM probe X′, 1 μM probe Y′ and a certain amount of target DNA. The reaction mixture was firstly heated for denaturation at 95 °C for 3 min, and then 2 U of thermal Ampligase was added into the reaction mixture to perform the ligation reaction at 75 °C. The LCR reaction was carried out with 30 thermal cycles at 95 °C for 1 min and 46 °C for 1 min.

### Exo I and Exo III treatment

After tricyclic LCR, 50 U of Exo I, 50 U of Exo III and 10× NEBuffer I were added into the reaction mixture to digest the excess probes X, X′, Y′, and X′Y′ products by incubation at 37 °C for 30 min. The digestion reaction was terminated by incubation at 90 °C for 10 min, and stored at 4 °C.

### Gel electrophoresis

The reaction products of tricyclic LCR were analyzed with 15% denaturating polyacrylamide gel electrophoresis (PAGE) in 1× TBE buffer (9 mM Tris–HCl, pH 7.9, 9 mM boric acid, 0.2 mM EDTA) at a 110 V constant voltage for 50 min at room temperature. The gel was stained with a silver staining kit (81104-1000, Tiandz Inc., Beijing, China) and visualized by a Bio-Rad ChemiDoc MP Imaging System (Hercules, CA, USA).

### Hybridization reaction

The hybridization reaction was carried out in a buffer solution containing 100 mM Tris–HCl, 10 mM (NH_4_)_2_SO_4_, and 3 mM MgCl_2_, pH 8.0. The Cy5-labeled reporter probes, biotinylated capture probes and the XY products were incubated at room temperature for 20 min to obtain the sandwich hybrids (the molar ratio of capture probes to reporter probes was kept at 1 : 1). After hybridization reaction, the sandwich hybrids were assembled onto the surface of 605QDs through specific biotin–streptavidin interaction to form the 605QD–oligonucleotide–Cy5 nanostructures.

### Steady-state fluorescence measurements

The fluorescence signals of reaction products were measured by an F-7000 spectrometer (Hitachi, Japan) with an excitation wavelength of 488 nm. The emission spectra were scanned from 500 to 800 nm, and the emission intensities at 605 nm (the maximum emission of 605QDs) and 670 nm (the maximum emission of Cy5) were used for data analysis.

### Total internal reflection fluorescence (TIRF)-based single-molecule detection

The reaction products were diluted 100-fold in the imaging buffer (1 mg mL^–1^ glucose oxidase, 0.4% (w/v) d-glucose, 0.04% mg mL^–1^ catalase, 50 μg mL^–1^ BSA, 67 mM glycine–KOH, 1 mg mL^–1^ trolox, 2.5 mM MgCl_2_, pH 9.4). For TIRF imaging, 10 μL of samples was directly pipetted to the coverslips. A sapphire 488 nm laser (50 mW, Coherent, U.S.A.) was used to excite the 605QDs. The photons from the 605QD and Cy5 were collected by a 100× objective (Olympus, Japan) and imaged with an exposure time of 100 ms by an Andor Ixon DU897 EMCCD. Generally, six frames of images from six different locations were acquired for every sample and a region of interest (200 × 400 pixels) of each image was selected for Cy5 molecule counting through the image J software.

### Cell culture and extraction of genomic DNA

Nonsmall-cell lung cancer cell line (H157 cells) was cultured in Roswell Park Memorial Institute (RPMI) Medium 1640 supplemented with 10% fetal bovine serum. Small-cell lung cancer cell line (H209 cells) was cultured in RPMI Medium 1640 supplemented with 20% fetal bovine serum. Both cell lines were cultured in a humidified incubator containing 5% CO_2_ at 37 °C. Genomic DNA was extracted by a universal genomic DNA extraction kit Ver. 5.0 (TaKaRa Biotechnology Co., Ltd., Dalian, China) according to the manufacturer’s instruction. Genomic DNA was fragmented through Pst I and BstE II digestion for 60 min at 37 °C, and subsequently subjected to cellular DNA methylation assay.

## Conflicts of interest

There are no conflicts to declare.

## Supplementary Material

Supplementary information
