# Correction: Single quantum dot-based nanosensor for sensitive detection of 5-methylcytosine at both CpG and non-CpG sites

**DOI:** 10.1039/c8sc90012d

**Published:** 2018-01-31

**Authors:** Zi-yue Wang, Li-juan Wang, Qianyi Zhang, Bo Tang, Chun-yang Zhang

**Affiliations:** a College of Chemistry , Chemical Engineering and Materials Science , Collaborative Innovation Center of Functionalized Probes for Chemical Imaging in Universities of Shandong , Key Laboratory of Molecular and Nano Probes , Ministry of Education , Shandong Provincial Key Laboratory of Clean Production of Fine Chemicals , Shandong Normal University , Jinan 250014 , China . Email: cyzhang@sdnu.edu.cn ; Fax: +86-0531-82615258 ; Tel: +86-0531-86186033; b Nantou High School Shenzhen , Shenzhen , 518052 , China

## Abstract

Correction for ‘Single quantum dot-based nanosensor for sensitive detection of 5-methylcytosine at both CpG and non-CpG sites’ by Zi-yue Wang *et al.*, *Chem. Sci.*, 2018, DOI: ; 10.1039/c7sc04813k.



## 

The authors regret that the incorrect Fig. 3 and the incorrect TOC entry were included in the original manuscript. The correct Fig. 3 is presented herein and an updated TOC entry accompanies the manuscript on the platform.

**Fig. 3:**
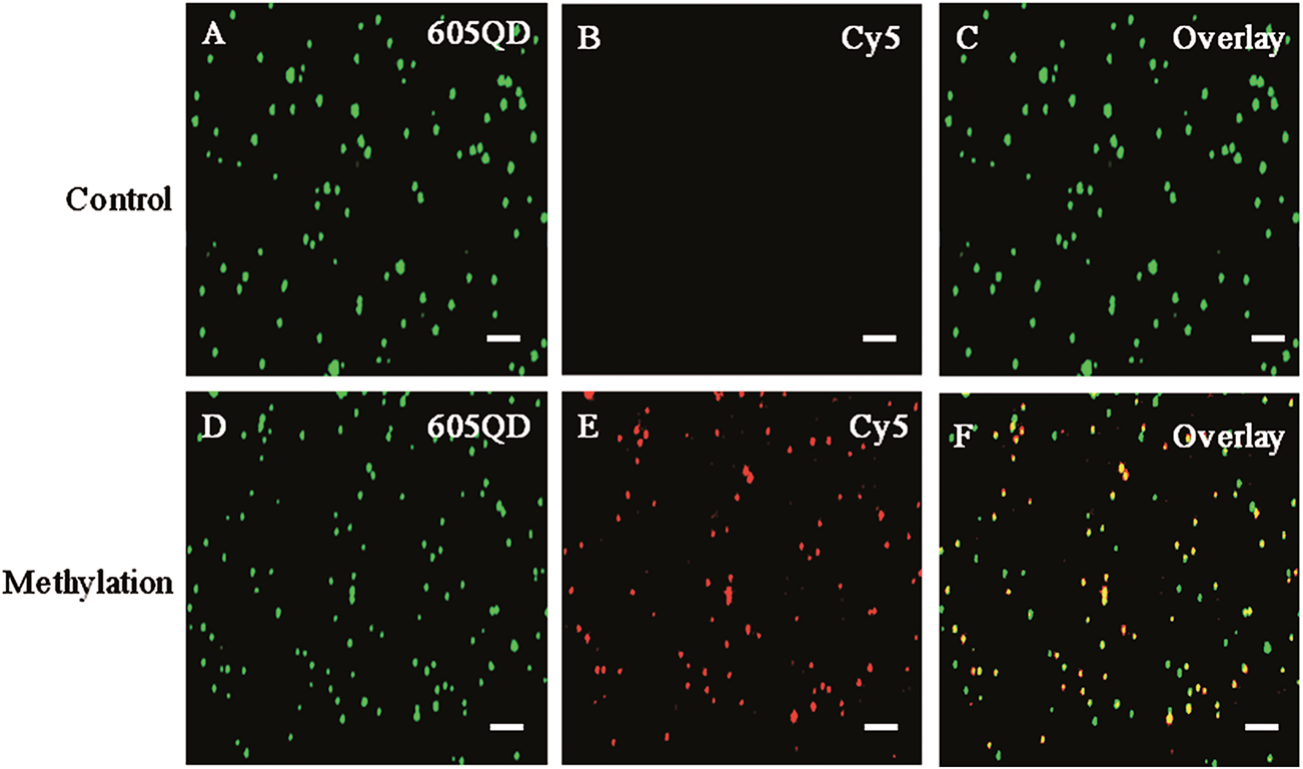
Fluorescence images of 605QD and Cy5 obtained by TIRF-based single-molecule detection in the absence (A–C) and presence (D–F) of methylated DNA. The fluorescence signal of 605QD is shown in green (A and D), and the fluorescence signal of Cy5 is shown in red (B and E), and the colocalization of the 605QD and Cy5 fluorescence signals is shown in yellow (C and F). The concentration of methylated DNA is 1.0 ×10^–11^ M. The concentration of each DNA probes X, Y, X′ and Y′ is 1.0 × 10^–6^ M. The concentration of 605QD is 8.3 nM. The scale bar is 2 μm.

The Royal Society of Chemistry apologises for these errors and any consequent inconvenience to authors and readers.

