# Supplementary material for: Single quantum dot-based nanosensor for sensitive detection of 5-methylcytosine at both CpG and non-CpG sites
Source: Chem Sci. 2017 Dec 13;9(5):1330–8. doi: 10.1039/c7sc04813k (PMC5887231; doi:10.1039/c7sc04813k)
Supplement: Supplementary file 1 [file SC-009-C7SC04813K-s001.pdf]

Electronic Supplementary Material (ESI) for Chemical Science.
This journal is © The Royal Society of Chemistry 2017

Electronic Supplementary Information

Single quantum dot-based nanosensor for sensitive detection of 5-methylcytosine at both CpG and non-CpG sites

Zi-yue Wang,[#,†] Li-juan Wang,[#,†] Qianyi Zhang,[‡,†] Bo Tang,[#,*] and Chun-yang Zhang[#,*]

[#]College of Chemistry, Chemical Engineering and Materials Science, Collaborative Innovation Center of Functionalized Probes for Chemical Imaging in Universities of Shandong, Key Laboratory of Molecular and Nano Probes, Ministry of Education, Shandong Provincial Key Laboratory of Clean Production of Fine Chemicals, Shandong Normal University, Jinan 250014, China

[‡] Nantou High School Shenzhen, Shenzhen, 518052, China

* Corresponding author. Tel.: +86 0531-86186033; Fax: +86 0531-82615258. E-mail: cyzhang@sdnu.edu.cn. Tel.: +86 0531-86180010; Fax: +86 0531-86180017; tangb@sdnu.edu.cn.

1. Optimization of thermal ampligase concentration

The thermal ampligase is responsible for the successful performance of tricyclic LCR amplification. We optimized the thermal ampligase concentration. As shown in Figure S1, the value of $F-F_0$ increases with an increasing concentration of ampligase from 0.025 to 0.05 U/μL (F is the Cy5 fluorescence intensity in the presence of methylated DNA target, and F_0 is the Cy5 fluorescence intensity in the absence of methylated DNA target), but decreases beyond the concentration of 0.05 U/μL. Thus, 0.05 U/μL ampligase is used in the subsequent experiments.

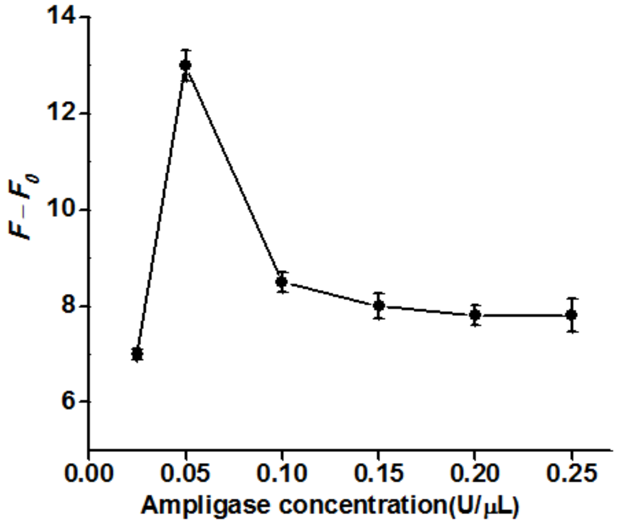

Figure S1. Variance of $F-F_0$ value with the concentration of methylated DNA. Error bars show the standard deviations of three independent experiments.

2. Optimization of ligation temperature

We further optimized the ligation temperature of LCR. As shown in Figure S2, the value of $F-F_0$ increases with the increasing reaction temperature from 42 to 46 °C, but decreases beyond 46 °C. Thus, the temperature of 46 °C is used in the subsequent experiments.

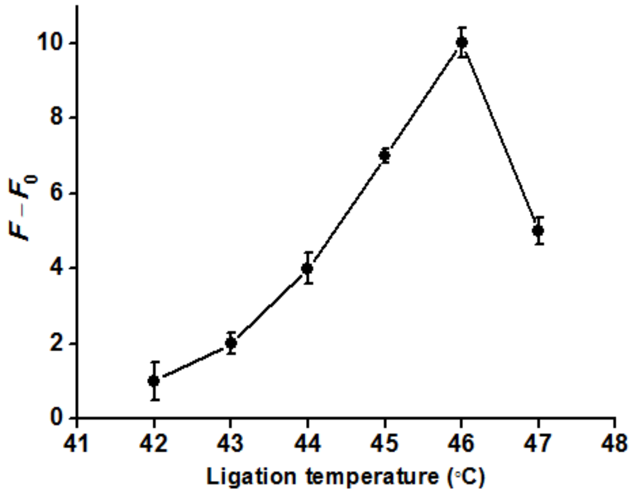

Figure S2. Variance of $F{-}F_0$ value with the ligation temperatures. Error bars show the standard deviations of three independent experiments.

3. Optimization of reaction cycle number

The thermal cycle number is directly related to the amplification efficiency of LCR. As shown in Figure S3, the value of $F{-}F_0$ increases with the increasing thermal cycle number from 20 to 30 cycles, but decreases beyond the cycle number of 30 cycle. To ensure the high sensitivity and good specificity, the thermal cycle number of 30 cycles is used in the subsequent experiments.

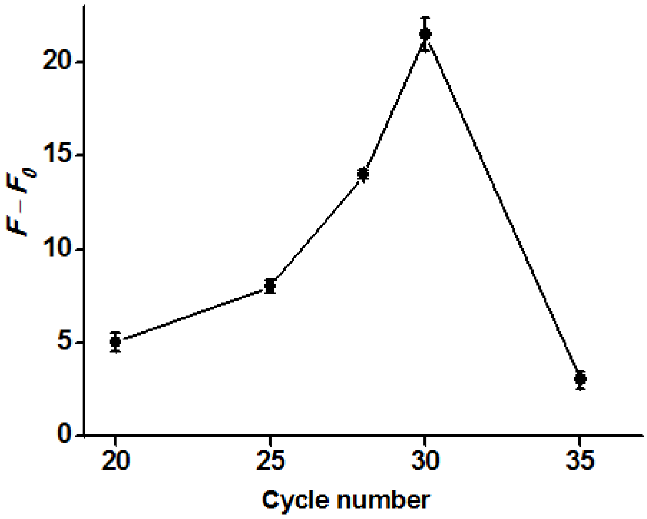

Figure S3. Variance of $F{-}F_0$ value with the thermal cycle number. Error bars show the standard

deviations of three independent experiments.

4. Optimization of the ratio of Cy5-labeled capture probe to the 605QD

To optimize the Cy5-labeled capture probe-to-605QD ratio, we synthesized a DNA sequence that is same to the ligation product of capture and reporter probes. The FRET efficiency (E) is calculated based on equation 1:

$$E=1-F_{DA}/F_{D} \qquad (1)$$

where F_{DA} is the 605QD fluorescence intensities in the presence of Cy5-labeld capture probes, and F_{D} is the 605QD fluorescence intensities in the absence of Cy5-labeld capture probes. As shown in Figure S4, both the FRET efficiency and the Cy5 fluorescence intensity increase with the increasing Cy5-labeled substrate-to-QD ratio from 12/1 to 48/1, and level off at the ratio of 24/1. Therefore, 24/1 is used for the ratio Cy5-labeled capture probe-to-QD in the subsequent experiments.

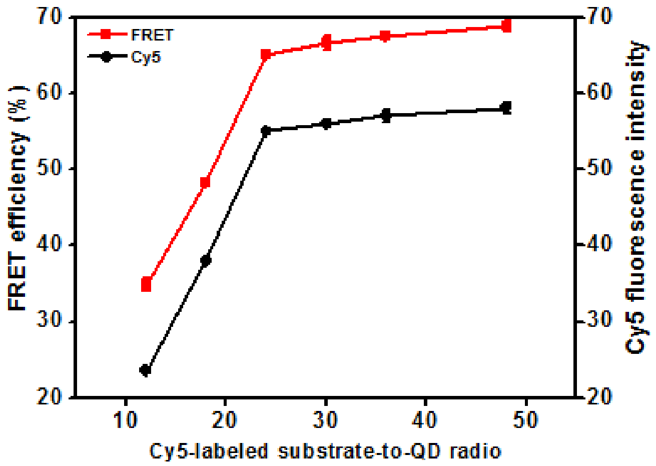

Figure S4. Variance of Cy5 fluorescence intensity (black curve) and FRET efficiency (red curve) with the Cy5-labeled substrate-to-QD ratio. The concentration of 605QD is 0.83 nM. Error bars show the standard deviation of three experiments.

5. Detection of DNA methylation level in the mixture

We further investigated the variance of fluorescence spectra with the input methylation level in the mixture of methylated and unmethylated DNA. As shown in Figure S5, with the increase of methylated DNA amount, the fluorescence intensity of 605QD decreases gradually, and the fluorescence intensity of Cy5 increases correspondingly, indicating efficient FRET from the 605QD to Cy5 induced by methylated DNA. Therefore, this method can be used to detect DNA methylation level in the mixture of methylated and unmethylated DNA.

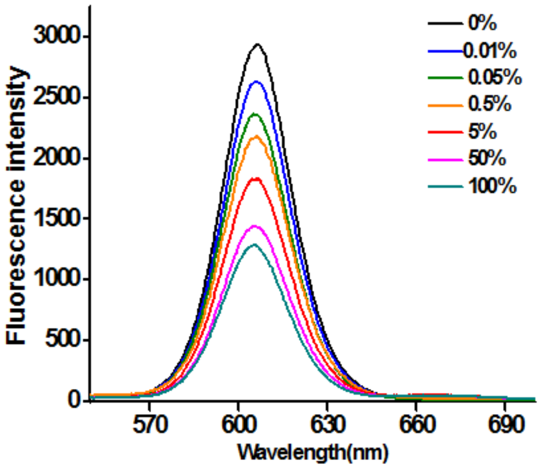

Figure S5. Variance of fluorescence spectra with the input methylation level in the mixture of methylated and unmethylated DNA. The concentration of 605QD is 8.3 nM.

6. Detection of Cellular DNA Methylation Level.

We measured the fluorescence spectra of same cellular samples in Figure 6. As shown in Figure S6A, a high Cy5 signal is obtained in H157 cells (Fig. S6A, red line), but no significant Cy5 signal is observed in H209 cells (Fig. S6A, blue line) and control group with only lysis buffer (Fig. S6A, black column), respectively, consistent with the results obtained by single QD-based nanosensor (Figure 6A). Moreover, the Cy5 signal improves with the increasing number of H157

cells in the range from 1 to 10000 cells (Fig. S6B), consistent with the result obtained by single QD-based nanosensor (Figure 6B).

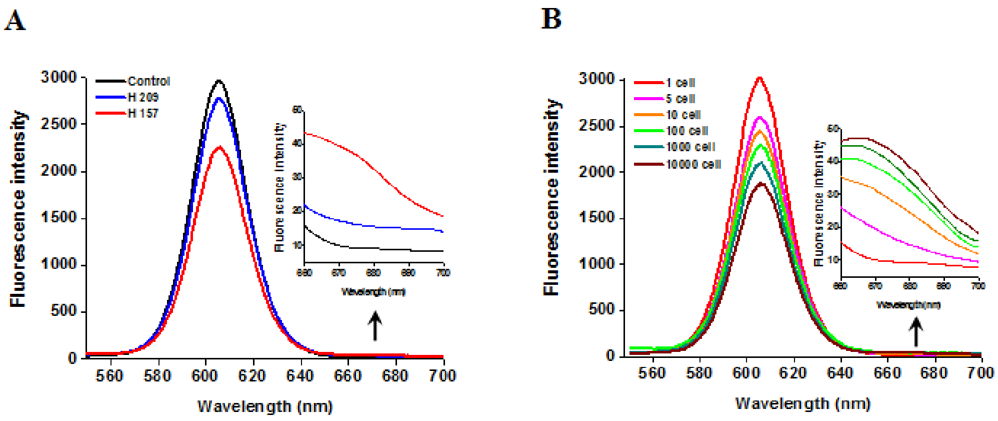

Figure S6. (A) Measurement of fluorescence spectra in the presence of lysis buffer (control group, black curve), 1000 H209 cells (blue curve), and 1000 H157 cells (red curve), respectively. Inset shows the magnified fluorescence spectra from 660 to 700 nm. (B) Measurement of fluorescence spectra in response to different number of H157 cell in the range from 1 to 10000 cells. Inset shows the magnified fluorescence spectra from 660 to 700 nm. The concentration of each DNA probes X, Y, X′ and Y′ is 1.0×10^{-6} M. The concentration of 605QD is 8.3 nM.

7. Sequences for detecting DNA methylation level in cancer cells

Table S1. Sequences of the Oligonucleotides[α]

note	sequences (5'-3')
probe X1	CGC CGA GCG CAC GCG
probe Y1	P-GTC CGC CCC ACC CTC **AG**
probe X′1	CTG AGG GTG GGG CGG AC
probe Y′1	P-CGC GTG CGC TCG GCG
capture probe1	CGC GTG CGC TCG GCG-biotin
reporter probe1	TGA CTG AGG GTG GGG CGG AC**T**

[α] In probe Y 1, the "P" presents the phosphate group (PO_4) modification at the 5′ end, and the bold "AG" bases present the phosphorothioate (PS) modification at the 3′ end. In probe Y′ 1, the "P" indicates the PO_4 modification at the 5′ end. In capture probe 1, the 3′ end is modified with a biotin. In reporter probe 1, the bold "T" base is modified with a Cy5 molecule.

8. Comparison of reported methods for DNA methylation assay.

Table S2. Comparison of various methods for DNA methylation assay.

strategy	method	requirement of specific sequence	involvement of polymerase	detection of non-CpG sites	LOD[a]	ref.
restriction cleavage	PCR-based QCM[b] detection	+	+	—	20 nM	1
restriction cleavage	GO[c]-based electrochemical detection	+	—	—	0.1 pM	2
bisulfite conversion	NESA[d]-based fluorescence detection	+	+	—	0.78 pM	3
ligation	HRCA[e]-based fluorescence detection	+	+	—	0.8 fM	4
ligation	PCR-based gel analysis	—	+	+	20 aM	5
ligation	LCR-based QD-mediated FRET	—	—	+	1 aM	this work

[a] LOD, limit of detection, [b] QCM, quartz crystal microbalance, [c] GO, graphene oxide, [d] NESA, nicking enzyme signal amplification, [e] HRCA, hyperbranched rolling circle amplification. Note: some methods for DNA methylation assay are not listed in this table because they only provide qualitative data instead of quantitative data.

References

1. J. Wang, Z. Zhu and H. Ma, *Anal. Chem.*, 2013, **85**, 2096-2101.
2. W. Li, P. Wu, H. Zhang and C. Cai, *Anal. Chem.*, 2012, **84**, 7583-7590.
3. A. Cao and C.-y. Zhang, *Anal. Chem.*, 2012, **84**, 6199-6205.

4. G. Zhu, K. Yang and C.-y. Zhang, *Biosens. Bioelectron.*, 2013, **49**, 170-175.

S. Rao, Y. Chen, T. Hong, Z. He, S. Guo, H. Lai, G. Guo, Y. Du and X. Zhou, *Anal. Chem.*, 2016, **88**, 10547-10551.
